# A Case of Polydipsia Complicated with Chronic Spasmodic Action of the Stomach

**Published:** 1883-02

**Authors:** D. D. Marr

**Affiliations:** Chesterton, Ind.


### Article IV.

### A Case of Polydipsia, Complicated with Chronic Spasmodic Action of the Stomach. By D. D. Marr, m.d., Chesterton, Ind.

Mrs. W., farmer's wife, aged forty years, mother of several children, complexion light, disposition rather hysterical—has been an invalid for ten years, the last three passing most of the time in bed. Weight, 115 pounds; very nervous, and has the general appearance of continued suffering.

She was quite intelligent, and informed me that she had been affected with the diseases for which she had called me to treat for three years, with but slight remissions.

During this time she had had several medical attendants to examine and treat her, no two of them agreeing as to their diagnosis, and none of them affording any relief. The last was a respectable practitioner of fifty years, who, after several visits, informed her that he was yet ignorant of her diseases, and discontinued his treatment.

On examination, I found her pulse 115, small and thready; appetite very limited, bowels constipated, tongue not much coated, but dry and fissured; great thirst, drank from one to two gallons of water per day, and passed urine in large quantities; sp. gr. 1,008, which, on several examinations, contained no sugar.

I was not a little surprised to hear gurgling, plashing and whirring sounds of confined liquid issuing from the region of her stomach, and with such violent action as to jerk and agitate her whole body, which she was unable to restrain.

On uncovering her abdomen, nothing unusual as to contour was noticed, excepting a slight fullness over the gastric region. Within the abdominal wall, and in the region of the stomach, and, in fact, the stomach itself, were all manner of writhings, twistings, and contractions in connection with the liquid sounds I have described.

Thinking that I could restrain the movements, I applied my hands firmly in various positions over the stomach, but with only partial success. I could very distinctly feel the contortions of the stomach and movements of the liquids therein.

She would frequently drink a half-pint of water, and immediately the movements and liquid sounds I have described would increase vigorously, and with great discomfort to the patient.

One homœopathic physician had diagnosed three or four snakes in her stomach, and prescribed fasting, so that the reptiles would hunger and crawl out.

Diagnosing her disease according to the heading of this article, I administered treatment as the indications required. Her hygienic and dietetic conditions were put in as favorable condition as her humble circumstances would permit, and tonics were freely given. Opium and its preparations were given even to a degree of narcotism, relieving the pain, but not the spasmodic action of the stomach. Anti-spasmodics had no appreciable benefit. Bismuth sub. nit., in drachm doses, three or four times daily was the only remedy that gave any relief, partially controlling the spasmodic action and allowing more ingestion of food. With a view of giving quietness and rest to the stomach, rectal alimentation was resorted to, but with no material benefit, as the constant desire for liquids could not be supplied per rectum.

She passed out of my observation for three or four months, when she again sent for me. She informed me that she had improved considerably, and that she could take nourishment and digest it with more comfort and less spasmodic action of the stomach, but that the disease was "moving downward."

On examination, I found the lower region of the abdomen enlarged, and she stated that there was movement connected with the enlargement. On examination, I found this due to pregnancy, which, in due time, terminated favorably. After the termination of gestation her condition improved, the spasmodic action of the stomach was but slight, and the quantity of fluids ingested was greatly lessened.

I have searched the medical literature in my possession, but have failed to find a reported case of chronic spasmodic action of the stomach.

May not the falsely reported cases of movements of snakes, lizards, and other reptiles in the stomach be due to a chronic spasmodic action of this organ, as in this instance. If so, we can easily explain away such reports. In this case, the continual drinking of large quantities of liquids was undoubtedly a great factor in the spasmodic action of the organ.

---

THE Hungarian medical journal, *Gyogyaszat*, publishes an interesting but not very æsthetical extract from an old materia medica for the year 1564. This materia medica appeared under the title: *Connumeratio portarum cottus Saladiensis anni* 1564, *Summa facit portas turcis non subjectas No.* 89¼. *Turcis vero subjectas No.* 364¼. Among the prescriptions are the following: Against toothache, powder the tooth of a hanged man, mix it with lard, and rub the aching tooth; it will fall out immediately. Against jaundice: Wash your posteriors very cleanly, and give the patient the water to drink before breakfast. Against stone in the bladder: Give the patient seven living lice in honey at the time of the declining moon. The whole extract shows the ignorance of the public and the learned in those times, but there are now prescriptions used which will be ridiculed a hundred years after us.

